# An Antibody-Based Leukocyte-Capture Microarray for the Diagnosis of Systemic Lupus Erythematosus

**DOI:** 10.1371/journal.pone.0058199

**Published:** 2013-03-13

**Authors:** Ming-Wei Lin, Joshua W. K. Ho, Leonard C. Harrison, Cristobal G. dos Remedios, Stephen Adelstein

**Affiliations:** 1 Department of Clinical Immunology, Royal Prince Alfred Hospital, Sydney, New South Wales, Australia; 2 Discipline of Medicine, Sydney Medical School, The University of Sydney, Sydney, New South Wales, Australia; 3 Division of Genetics, Department of Medicine, Brigham and Women's Hospital, Harvard Medical School, Boston, Massachusetts, United States of America; 4 The Walter and Eliza Hall Institute of Medical Research, Department of Clinical Immunology and Burnet Clinical Research Unit, The Royal Melbourne Hospital, Parkville, Victoria, Australia; 5 Discipline of Anatomy, Bosch Institute, The University of Sydney, Sydney, New South Wales, Australia; Pavillon Kirmisson, France

## Abstract

The diagnosis of Systemic Lupus Erythematosus (SLE) is challenging due to its heterogeneous clinical presentation and the lack of robust biomarkers to distinguish it from other autoimmune diseases. Further, currently used laboratory tests do not readily distinguish active and inactive disease. Several groups have attempted to apply emerging high throughput profiling technologies to diagnose and monitor SLE. Despite showing promise, many are expensive and technically challenging for routine clinical use. The goal of this work is to develop a better diagnostic and monitoring tool for SLE. We report a highly customisable antibody microarray that consists of a duplicate arrangement of 82 antibodies directed against surface antigens on peripheral blood mononuclear cells (PMBCs). This high-throughput array was used to profile SLE patients (n = 60) with varying disease activity, compared to healthy controls (n = 24), patients with rheumatoid arthritis (n = 25), and other autoimmune diseases (n = 28). We used a computational algorithm to calculate a score from the entire microarray profile and correlated it with SLE disease activity. Our results demonstrate that leukocyte-capture microarray profiles can readily distinguish active SLE patients from healthy controls (AUROC = 0.84). When combined with the standard laboratory tests (serum anti-dsDNA, complements C3 and C4), the microarrays provide significantly increased discrimination. The antibody microarrays can be enhanced by the addition of other markers for potential application to the diagnosis and stratification of SLE, paving the way for the customised and accurate diagnosis and monitoring of SLE.

## Introduction

Systemic Lupus Erythematosus (SLE) is an immune-mediated, multisystem, inflammatory disease characterised by autoantibody production. Its diagnosis relies on identification of combinations of clinical features and laboratory tests to distinguish it from other autoimmune disorders [1]. This is often problematic in clinical practice as some of the features required to fulfill the diagnostic criteria may take years to develop and some individuals with pathognomonic features do not meet all the established criteria. The clinical course of the disease is typified by unpredictable flares manifest by the onset of new organ involvement, worsening of existing disease, and periods of remission. Although several laboratory measurements, such as serum complement C3 and C4 levels, anti-dsDNA antibody titers and erythrocyte sedimentation rate (ESR) are routinely used in the clinic to help with disease management, individually they are not diagnostic of SLE and do not on their own give an accurate indication of disease activity. Consequently, assessment of disease activity and response to therapy remains largely clinical. The development of reliable biomarkers would enable us to distinguish between SLE and other autoimmune or infective conditions with similar clinical presentations, and would assist in the diagnosis and management of the condition. Such markers would ideally stratify the condition, predict flares, determine disease severity and activity, and response to therapy, and thereby limit unnecessary investigation and exposure to the side effects of immunosuppressive agents.

By using gene transcripts grouped into modules, Chaussabel *et al*. [2] developed a bioinformatics system to monitor disease activity in SLE. Their results raise the possibility of more precise characterisation of disease activity based on the pathogenesis of the condition and its molecular expression. However, the high cost, need for batch analysis and the delays in acquisition of results of molecular analyses using current technology limits the clinical utility of this approach for individual patients.

Autoantibodies are the hallmark of many autoimmune diseases. To circumvent the limitations of the DNA gene array, many groups [3]–[6] have employed miniaturised formats of parallel detection of autoantibodies using antigen microarrays. Of particular interest, Robinson *et al*. [4] fabricated 1152-feature arrays containing 196 distinct biomolecules representing major autoantigens targeted by autoantibodies from patients with autoimmune rheumatic diseases and found them to be of comparable sensitivity and specificity to conventional assays. Fattal *et al*. [7] used an array to measure antibodies to 930 different antigens including viral proteins in 40 SLE patients and successfully identified particular combinations of markers specific for the disease at various levels of activity. They quoted a sensitivity of >93% and specificity of >88% in distinguishing SLE from normal controls.

Belov *et al*. demonstrated the utility of leukocyte-capture antibody microarrays in the diagnosis, monitoring and stratification of patients with lymphoproliferative diseases [8], [9]. The concept was co-invented by one of the authors (CGdR) and has been successfully employed to identify different clonally expanded leukocytes populations that are characteristic of human leukaemias [8], [9]. However, it soon became clear that these cell capture microarrays could also be used to detect more subtle changes in leukocyte populations, for example cell activation or inflammatory changes in patients with heart disease and other inflammatory diseases [10]–[15]. We postulated that inflammatory diseases such as SLE would also cause changes to the expression of leukocyte surface molecules that could be detected by the cell array. To demonstrate the potential utility of this microarray in the diagnosis and stratification of SLE, we conducted a study of patients with SLE and demonstrated that the leukocyte capture array profile of these patients is readily distinguishable from that of healthy controls. By generating an immunophenotypic fingerprint for each patient, the cell capture microarray can identify common patterns that permit identification of subgroups based on disease activity. Notably, we show that our microarray can on its own distinguish SLE from healthy controls and can complement standard laboratory tests to improve stratification of SLE patients based on disease activity. Further customisation with other antibodies, based on knowledge of leukocytes surface antigen expression related to pathogenesis of SEL, is likely to improve the sensitivity and specificity of the array.

## Methods

### Microarray construction

Antibody microarrays (DotScan^TM^) were purchased from Medsaic Pty. Ltd., Sydney. The construction of the antibody microarray has been described previously [8]. It consists of a duplicate set of 82 mouse monoclonal antibodies directed against human cluster of differentiation (CD) antigens that are robotically placed on the surface of a nitrocellulose-coated glass slide (Schleicher and Schuell, FAST slides). The slides and the antibodies, including the appropriate isotype control antibodies, are listed in Figure S1. Unoccupied binding sites on the remaining surface of the slide are then blocked using powdered skim milk to minimise non-specific binding. A set of mouse isotype control antibodies is included, and the perimeter of the CD antibody array is marked by anti-CD44, a pan-leukocyte CD antibody, used to show that leukocytes are evenly distributed across the antibody area. Only 10 nL of each antibody is required for each spot, which, once deposited on the nitrocellulose surface, spreads to a diameter of 450–500 µm. This is large enough to accommodate up to 1500 leukocytes, providing an acceptable statistical sampling of the population of cells applied to the array surface. After 30 minutes any leukocytes that are not immobilized at the antibody dots are removed by gentle washing, the slide is scanned, and the attached cells are quantified by light scattering [8]. We previously reported a good correlation between the dot intensities on antibody microarrays and flow cytometry with the same antibodies [8], but both the production of the microarrays and the nitrocellulose slides have changed since this was published. Accordingly, we have included new data from the batch of antibody microarrays to those used in the experiments report in this paper. PBMCs from a healthy blood donor were stimulated by overnight exposure to anti-CD3 monoclonal antibody and then applied to microarrays and quantified by densitometry as described above and also monitored by flow cytometry. The microarray spot densities for four randomly selected expressed antigens (CD25, CD69, CD71 and HLA-DR) are well matched by the cell numbers determined by flow cytometry from the same sample (Figure S2).

### Patients

Written informed consent was obtained from 117 subjects attending clinics of the Royal Prince Alfred Hospital. 137 assessments were performed: 60 on patients with SLE, 53 with other autoimmune diseases including rheumatoid arthritis (RA) (N = 25), vasculitis (N = 9), polymyositis (N = 1), seronegative arthritis (N = 8), scleroderma (N = 3), Sjogren's syndrome (N = 1), undifferentiated connective tissue diseases (N = 6), and 24 healthy controls. All SLE patients fulfilled at least four of the American College of Rheumatologists (ACR) criteria for the disease [1] and their disease activity was determined according to the SLE Disease Activity Index (SLEDAI) score [16]. We assigned patients into three disease activity groups according to their SLEDAI score with ≥8 defined as clinically active, 5–7 as intermediate (semi-active) activity and, ≤4 as inactive. The details of their immunosuppressive medications were recorded at the time of each clinic visit. Thirty three (55%) patients had inactive disease, 16 (27%) had intermediate disease activity, usually following treatment of a disease flare, and 11 (18%) were clinically active. There were no differences between the demographics of the patients in any of the clinical categories, their use of immunosuppressants at the time of study or the number of ACR criteria fulfilled during the course of their illness (Table 1). The results of anti-dsDNA antibodies (Farr assay, Trinity Biotech, Ireland) and serum complements C3 and C4 levels (Immage® 800, Beckman Coulter) were recorded at each visit. The project was approved by the Royal Prince Alfred Hospital Ethics Committee (Approval number X06-0089).

**Table 1:** Demographic and clinical characteristics of the patients.

	SLE active	SLE intermediate	SLE inactive
Number	11	16	33
Demographics			
Age, mean (range), years	43 (36–59)	36 (28–48)	47 (24–80)
Female (%)	10/11 (91%)	14/16 (88%)	29/33 (88%)
Caucasian	8 (73%)	11 (69%)	26 (79%)
Asian	3 (27%)	5 (31%)	7 (21%)
No of ACR criteria fulfilled	6	6	7
Laboratory criteria			
Anti dsDNA positive*	10(91%)	9 (56%)	8 (24%)
C4*	7 (64%)	7 (44%)	7 (21%)
C3*	7 (64%)	10 (63%)	8 (24%)
Medications			
No immunosuppressants	3(27%)	2 (13%)	10(30%)
Prednisone alone	1(9%)	3 (19%)	7 (21%)
Immunosuppressants alone	2(18%)	1 (6%)	3 (9%)
Prednisone+immunosuppressants	5(45%)	10 (63%)	13(39%)

* at time of assessment

### Blood processing and microarray profiling

Anticoagulated whole blood was collected in EDTA tubes from each participant and maintained at room temperature until use within 24 hours. Leukocytes from peripheral blood mainly consisting of peripheral blood mononuclear cells (PBMCs) were isolated using Histopaque (Sigma-Aldrich), washed in PBS, resuspended in PBS containing 1 mM EDTA to a density of 10^7^ cells/ml, and incubated on the cell array for 30 min at room temperature; unbound cells were then gently washed off with PBS. Arrays were then fixed for at least one hour in PBS containing 1% (v/v) formaldehyde (Sigma-Aldrich) washed in PBS, scanned, and the light scattering by the attached cells recorded in an image file. The microarray data is accessible at National Center for Biotechnology Information (NCBI)'s Gene Expression Omnibus (Accession number: GSE27293).

### Preprocessing of microarray expression data

The raw intensity light scatter values at each spot on the slide were scale-normalised such that the expression value for each CD antigen is between 0 and 10. This normalisation method has been shown to generate antigen expression levels that correlate linearly with corresponding cell counts by flow cytometry [11]. Further, we applied two filtering criteria to the data: Firstly, antigens that had low expression across all samples were excluded. An antigen was deemed to have low expression if its median expression value among the 20% most highly expressed samples was less than 1, since this is approximately the lower limit of the technology to reliably detect antigen binding [11]. Secondly, CD antigens that had a low signal-to-noise ratio (SNR) were rejected. The SNR of an antigen was defined as the ratio of the standard deviation of its mean expression value across all samples against the mean absolute difference between the two intra-slide replicate measurements across all samples. CD antigens with a SNR less than 1.2 were filtered out since this ratio appeared to be a conservative threshold that filtered out most of the negative control spots on the array.

### Individual CD antigen biomarkers for SLE

To identify CD antigens that individually showed strong evidence of association with the class label (*e.g*., SLE or control), we performed a linear model analysis using the R package, *limma* [17], which is a program specifically designed for microarray analysis. A *p* value was calculated using the moderated *t* statistics for each CD antigen to determine whether it was statistically significant. We also calculated the Area Under the Receiver Operating Characteristic (AUROC) curve for each CD antigen. This analysis was performed using the R package *ROCR* [18].

We tested whether the expression levels of each individual CD antigen were significantly associated with SLE activity using a moderated *t* test and AUROC. Six sets of comparisons were performed to assess whether each individual marker was good for diagnosis or stratification of SLE. A CD antigen qualified as a singleton biomarker if it had p<0.05 and AUROC>0.7 in any comparison.

### Measurement of SLE activity based on the multiple biomarkers

We used an advanced classification algorithm, Support Vector Machine (SVM) [19], [20] to create an SLE activity score from the entire antigen expression profile. We used the SVM implementation in the R package *e1071* with default parameters (kernel = radial basis kernel, cost = 1). Briefly, a SVM uses labelled training examples to construct a non-linear decision rule (*i.e.*, a decision boundary) that can best distinguish the expression profiles of SLE patients from those of healthy controls. We assumed there is a linear trend in SLE disease activity; therefore we trained the SVM model using profiles from healthy controls and active SLE patients only. Once the SVM model was trained we calculated an SLE activity measure, which we refer to as an S-score, for every expression profile, *x*, based on the profile's distance, *f(x)*, from the decision boundary as determined by the SVM classifier. The S-score is calculated by the following formula:
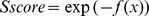



### Cross-validation

We used a three-fold repeated sub-sampling cross-validation strategy to assess the performance of the SLE activity score. Briefly, we iteratively performed the following steps 100 times: randomly partitioned the samples into three equal portions and used two of these portions as the training set to build a SVM model. The remaining portion was a testing set to assess the model's sensitivity and specificity for each of the three SLE activity states. An ROC curve and an AUROC value were generated at each iteration and the mean and standard deviation of AUROC of the 100 iterations were recorded.

## Results

### Analysis of singleton biomarkers for SLE

After the normalisation and filtering steps, 57 antibody spots passed our quality filters and were used in subsequent analyses. The results demonstrate (Table 2) that many T and NK-cell surface markers are down-regulated in SLE. Another important observation was that, although many of these CD antigens have statistically significant associations with the disease, none are good biomarkers of SLE activity because the AUROC values are not sufficiently high (<0.85), and do not positively correlate with disease activity. This indicates that no CD antigen alone, as measured by our antibody microarray, can be used as a reliable biomarker for monitoring the activity of SLE. We performed the same moderated *t* test and ROC analysis for each of the conventional laboratory markers (anti-dsDNA, complement C3 and C4), and found that they can generally differentiate patients with inactive SLE from those with active forms of SLE, but cannot reliably distinguish semi-active patients and active ones (Table 2). In particular, anti-dsDNA and C3 show reasonable discriminatory ability that no single marker in the current version of the microarray can match.

**Table 2:** A list of the CD antigens that exhibit statistically significant SLE-activity dependent expression patterns.

CD antigens	Category	Inactive vs. healthy		Semi-active vs. healthy		Active vs. healthy		Active vs. inactive		Semi-active vs. inactive		Active vs. semi-active	
		*p*	*AUROC*	*p*	*AUROC*	*p*	*AUROC*	*p*	*AUROC*	*p*	*AUROC*	*p*	*AUROC*
*Down regulated in SLE*													
TCR a/b	T cells	0.24	0.60	0.01	0.73	0.19	0.62	0.63	0.54	0.09	0.65	0.37	0.59
CD2	T cells	0.00	0.74	0.00	0.86	0.00	0.82	0.22	0.58	0.12	0.64	0.92	0.52
CD3	T cells	0.09	0.64	0.00	0.79	0.01	0.75	0.16	0.62	0.07	0.66	0.86	0.53
CD4	T cells	0.04	0.65	0.01	0.76	0.02	0.73	0.38	0.58	0.24	0.60	0.89	0.51
CD5	T cells	0.01	0.68	0.00	0.86	0.00	0.82	0.11	0.63	0.02	0.69	0.64	0.54
CD7	T cells	0.01	0.71	0.00	0.89	0.00	0.80	0.13	0.62	0.03	0.69	0.73	0.53
CD8	T cells	0.28	0.62	0.02	0.79	0.03	0.77	0.16	0.68	0.11	0.66	1.00	0.59
CD28	T cells	0.03	0.63	0.01	0.71	0.15	0.61	0.85	0.51	0.32	0.59	0.34	0.60
CD45RA	Naïve T and B cells	0.17	0.62	0.01	0.78	0.02	0.72	0.15	0.63	0.09	0.68	0.95	0.52
CD56	NK cells	0.00	0.67	0.00	0.78	0.01	0.75	0.57	0.58	0.26	0.59	0.71	0.50
CD57	NK cells	0.72	0.59	0.03	0.75	0.05	0.75	0.07	0.65	0.04	0.66	0.98	0.52
CD52	Membrane glycopeptide	0.01	0.70	0.00	0.82	0.06	0.67	0.99	0.51	0.35	0.58	0.48	0.59
kappa 1/4 IM	B cells	0.16	0.64	0.02	0.74	0.28	0.66	0.96	0.55	0.22	0.56	0.36	0.51
lambda 1/4 IM	B cells	0.22	0.63	0.05	0.68	0.15	0.70	0.58	0.56	0.30	0.57	0.76	0.50
*Up regulated in SLE*													
CD66c	Platelets	0.11	0.69	0.07	0.73	0.03	0.73	0.33	0.58	0.60	0.61	0.64	0.51
CD95	Granulocytes	0.30	0.59	0.76	0.52	0.04	0.70	0.16	0.64	0.22	0.61	0.03	0.74
*Conventional laboratory tests*													
dsDNA	dsDNA							0.01	0.80	0.12	0.70	0.22	0.62
C3	Complement C3							0.01	0.79	0.01	0.74	0.69	0.66
C4	Complement C4							0.54	0.65	0.11	0.69	0.49	0.57

Significant SLE-activity dependent expression patterns are determined based on moderated *t*-test and AUROC (*p*<0.05 and AUROC >0.7 in at least one comparison). All p-value less than 0.05 and AUROC value greater than 0.7 are highlighted in bold.

**Figure 1:**
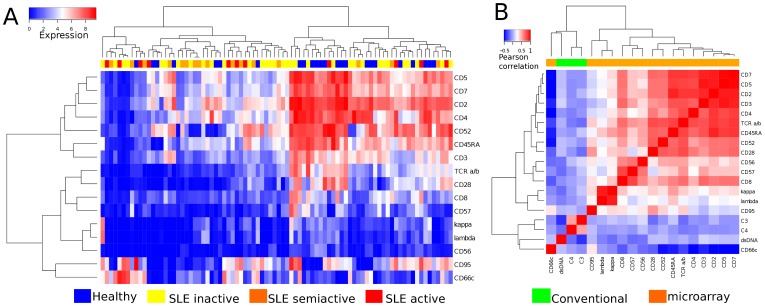
SLE singleton biomarker analysis. (A) Heat map of singleton CD biomarkers from SLE patients and healthy controls. (B) Heat map of Pearson's correlation coefficient between each pair of CD biomarkers and conventional laboratory biomarkers.

The heat map in Figure 1A shows the expression pattern of singleton CD antigen biomarkers. Hierarchical clustering reveals two large clusters of profiles, one dominated by SLE patients (left hand side of Figure 1A), and one dominated by healthy subjects (right side of Figure 1A). Although the clustering analysis cannot perfectly separate the SLE patients from healthy controls, the results suggest that the antigen profiles contain useful discriminatory information for building a diagnostic test.

To investigate whether the set of CD biomarkers contained redundancy (by having multiple biomarkers with highly correlated expression patterns), we performed a Pearson's correlation coefficient among the expression profile of all singleton CD antigen biomarkers listed in Table 2. Figure 1B shows that there are several strongly co-expressed clusters of CD antigens among T cell (CD7, CD5, CD3, and CD2), NK cell (CD56, CD57) and B cell markers (kappa, lambda). Some of the CD antibodies that produced highly correlated antigen expression profiles can therefore be selectively removed from the microarray without decreasing its predictive value. It is well known in the machine learning literature that the best feature sets for classification are those that individually are strongly associated with the class label, but are unrelated to each other. The conventional serum biomarkers such as C3, C4 and anti-dsDNA measured in the patient cohort have little correlation with other CD biomarkers in the study, suggesting that they can provide complementary information in the assessment of SLE.

### Leukocyte capture arrays can discriminate SLE from healthy subjects

**Figure 2:**
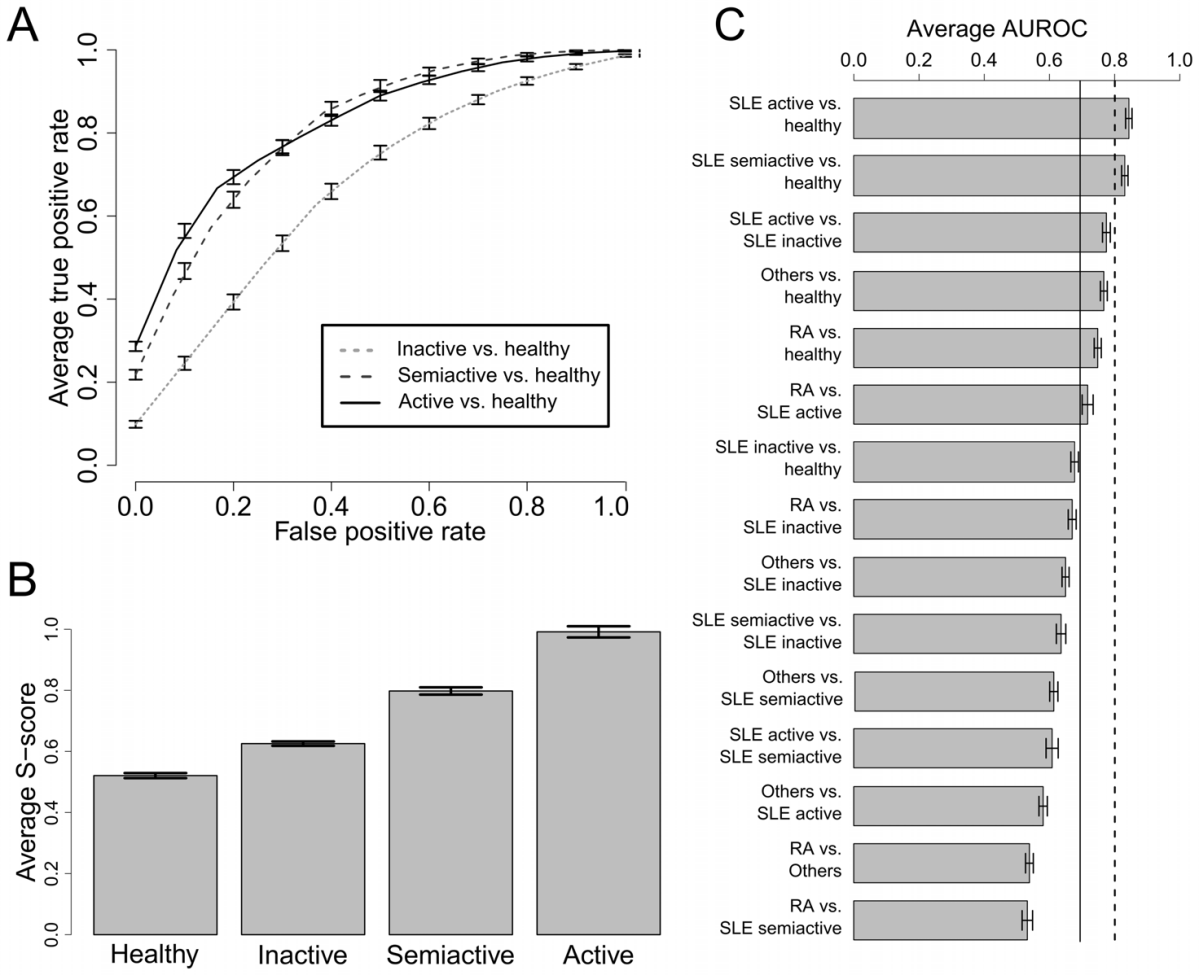
Cross-validation analysis of a SVM based classifier for diagnosis and stratification of SLE. **(A)** ROC analysis of the SLE classification measure. **(B)** The average S-score of test samples from 100 rounds of cross-validation (error bar represents S.E.M). **(C)** Average AUROC of comparisons between SLE, healthy controls, rheumatoid arthritis (RA) and other autoimmune diseases (others). The black dotted line at AUROC = 0.8 indicates a classifier that can readily separate the two classes, and a black solid line at AUROC = 0.7 indicates a classifier that is moderately effective for separating two classes (error bar represents S.E.M).

Next we explored whether building a SLE classification rule based on the expression of multiple antigens can result in a more accurate and robust diagnostic assay. We built a multivariate classifier for our microarray profile using SVM, and validated the classification performance using rigorous cross-validation. We used this SVM classification approach to construct a SLE diagnostic score, called the S-score, using expression of all the 57 CD antigens measured, and assessed the quality of the S-score using cross-validation. The ROC plots in Figure 2A demonstrate that inactive SLE cannot be reliably separated from healthy controls, but semi-active and active SLE patients can (AUROC = 0.83 and 0.84 respectively). In addition, we also investigated whether the S-score is positively correlated with SLE disease activity. The average S-score of the samples belonging to the four classes (healthy, SLE inactive, SLE semi-active and SLE active) from the 100 rounds of cross-validation are summarised in Figure 2B, and demonstrate a positive correlation between the S-score and disease activity. This is a particularly encouraging finding since the SVM model was trained using samples from healthy subjects and active SLE patients only, but it correctly identified the relative disease activity of semi-active and inactive patients, which supports the robustness of this approach.

We also investigated whether the antibody array can distinguish SLE patients from patients with other autoimmune diseases. In this analysis, we independently built a two-class SVM classifier for each comparison (RA vs. healthy, RA vs. SLE inactive, RA vs. SLE semi-active and so on) as shown in Figure 2C. These analyses show that active and semi-active SLE can be reliably distinguished from healthy controls with an AUROC greater than 0.8 (black dotted line). Active SLE compared to inactive disease, other autoimmune diseases and RA compared to healthy subjects and RA compared to active SLE, have AUROC values between 0.7 and 0.8 (between the black solid and dotted lines) that indicate discrimination with less certainty, while the remainder of the diseases with AUROC values <0.7 (to the left of the solid black line (*e.g.*, RA and other autoimmune diseases vs. SLE inactive etc) cannot be reliably distinguished.

### Leukocyte capture arrays improve the discriminative ability of conventional laboratory tests in SLE

**Figure 3:**
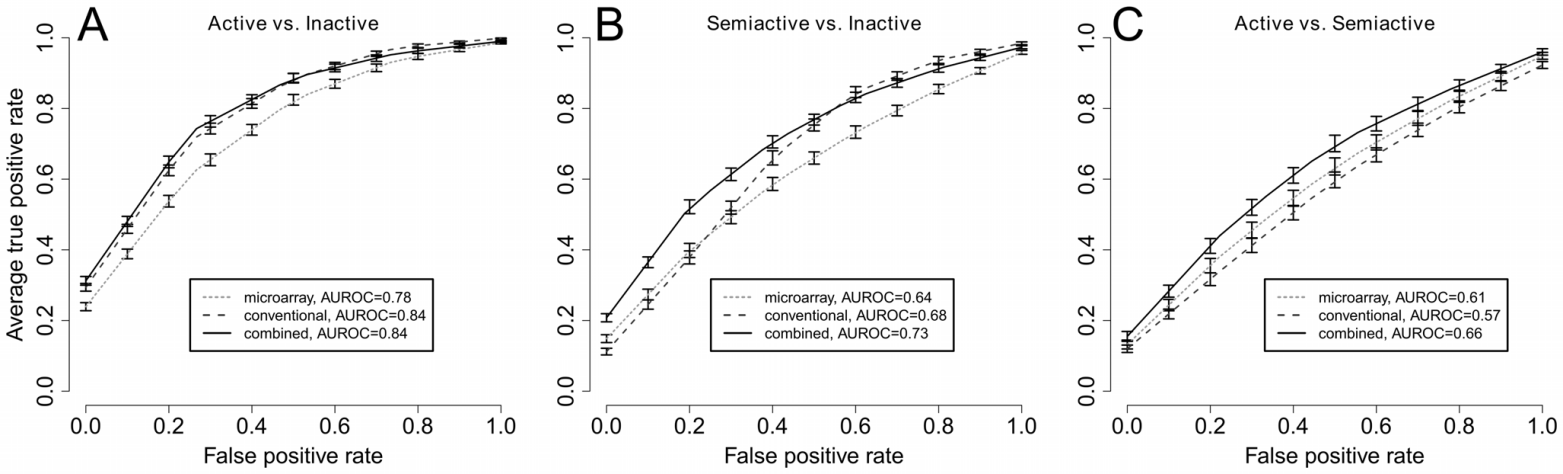
Comparison of discriminatory ability of CD antibody microarray and conventional laboratory tests.

Currently the assessment of SLE activity is based on a combination of clinical symptoms, signs, and laboratory tests. Since discriminating inactive SLE from more active forms of SLE (*i.e.*, semi-active or active) may be important in management, we evaluated the discriminatory ability of combination of tests including antibody arrays and the serological and immunochemical tests performed. For each of the datasets, we trained a classifier using the SVM approach, and its discriminatory ability was evaluated with 100 rounds of three-fold cross validation (Figure 3). The serum tests, anti-dsDNA antibodies, C3, and C4 levels, are more discriminating than our antibody microarray (*p*<0.001, one-sided paired *t* test) in distinguishing semi-active and active SLE from inactive SLE (Figure 3A and B). However, our antibody microarray can better distinguish active from semi-active SLE (*p*<0.001). When we trained a SVM based on the results of both microarray and the serum tests, we achieved a significantly better separation of semi-active SLE from inactive and active SLE (*p*<0.001 for both classification tasks). Given the clinically heterogeneous presentation of SLE patients, it is particularly encouraging to note that the microarray can improve the discriminative ability of conventional laboratory tests for semi-active vs. inactive SLE. Taken together with the observation that nearly all singleton CD biomarkers have very low expression correlation with the measurements from the three conventional laboratory tests (Figure 1B), we believe that the leukocyte microarray does indeed provide additional information.

## Discussion

This antibody microarray yields comparable diagnostic sensitivity and specificity to the DNA microarray system described by Chaussabel *et al*. [2]. The antibody microarray we described is, however, a potentially more practical platform for use in the diagnostic laboratory, since the analytical equipment required is less technically challenging and much less expensive (cost per microarray is ∼US$90) than gene expression microarrays or flow cytometry for the same number of antibodies, and it can be performed rapidly on an *ad hoc* basis. Traditionally, cellular markers and populations (and more importantly the changes in these populations) are detected by flow cytometry. This is generally a technically challenging, slow and expensive method that, when performed in a sophisticated laboratory with large sample volumes of sera and reagents and skilled technicians, can analyze no more than 17 CD antigens in a single analysis. The cell capture microarrays, in contrast to flow cytometry, can be performed on the bench in any laboratory and can simultaneously determine the presence of more than 100 antigens expressed on the surface of leukocytes. Furthermore, the microarray provides a semi-quantitative method of monitoring the numbers of cells in a sample since the light scattering from the cells is proportional to the number of cells immobilized on the arrays [8], [11]. In our experience, the method requires minimal training and uses only small volumes of antibodies and peripheral blood. In addition to the discriminatory ability demonstrated in this study, the technical simplicity of this profiling technology makes it attractive for clinical use.

The high-throughput nature of this microarray is also particularly attractive because it allows rapid identification of an immunophenotype for each patient based on the expression of multiple cell surface molecules that have been perturbed due to current or previous activation events in SLE. In principle, combinations of expressed surface membrane proteins (CD antigens and other membrane proteins) characterise functional subsets of leukocytes. One key idea of this study is that such leukocyte surface markers carry useful information about SLE activity. Our data represent a proof of concept only and will need to be validated using a much larger sample size before the microarray is applied in a clinical setting.

It is possible to turn our antibody microarray approach on its head, namely to immobilize the antigen and capture the antibodies as undertaken by Fattal *et al*. [7] and Robinson *et al.* [4]. Although these methods can distinguish SLE from healthy controls, their clinical performance in distinguishing SLE from other autoimmune and rheumatic has not been demonstrated and performance characteristics in the clinic are not yet reported. Our pilot study demonstrates that leukocyte surface markers contain information that may be used alone or in combination to other markers (such as anti-dsDNA) to distinguish SLE from normal controls, stratify disease activity, and may even distinguish SLE from other autoimmune and rheumatic conditions, although the later requires further study.

We show that a disease activity score can be constructed based on the entire antigen expression profile through a state-of-the-art machine learning technique. Similar supervised machine learning approaches for clinical diagnostic support using microarray technology have been reported extensively in the last decade, particularly in the field of cancer classification [21], [22]. Unlike a traditional singleton biomarker, the calculation of our S-score depends on the size and quality of the training examples. Different SVM decision functions are generated by using different training samples. Therefore, we performed a cross-validation analysis to estimate the sensitivity and specificity of this classifier for SLE. This analysis indicates that our approach for generating the S-score is generally robust even when different subsets of the training samples are used.

We note that some CD antigens, including CD95 [23], CD86 [24], [25], which have previously been reported to be associated with SLE, do not show statistical significance in our singleton biomarker analysis. This is surprising given their role in co-stimulation and cell activation. We suspect this may reflect the lack of specificity of the clone of antibodies used in this version of the microarray. From our experience, different clones of antibodies have different antigen-binding characteristics, and further experiments using different antibody clones will determine if alternative antibodies will contribute to the discriminatory ability of the array.

Although the discriminatory ability of the current version of the microarray on its own is no better than conventional tests, the fact that the microarray provides additional information for clinical assessment of SLE is an important finding. The expression values from about 40% of the antibody spots in the current version of the microarray were filtered out because of low expression or high variability in performance. This is not surprising as this version of microarray was originally designed for the diagnosis of lymphoproliferative diseases [8]. It is likely that a customised antibody microarray containing rationally selected antibodies that target CD antigens known to reflect the pathogenesis and activity of SLE will improve the sensitivity and specificity of the antibody microarray [26]. In particular, we propose that a customised SLE antibody microarray can be developed by retaining only those informative biomarkers in the current generation of the array, and augmenting the array with other antibodies directed to cell surface markers whose expression has been reported to be perturbed in patients with SLE. These could include antibodies related to innate immunity (CD1a, CD14, CD83), adaptive immunity (CD161), adhesion molecules (CD166 and CD6), other markers (CD279 and other chemokines *e.g.*, CCR6). These and other molecules have been shown to be potentially involved in the pathogenesis of SLE and may therefore also be useful markers of the disease and level of activity [26], [27].

## Conclusion

Our observations using a first-generation antibody microarray demonstrate that SLE patients can be distinguished from healthy controls based on the differential expression of CD markers on PBMCs. Our analysis indicates that the current version of PBMC CD antibody microarray can be combined with current laboratories tests to provide superior discriminatory ability to stratify SLE patients according to disease activity. We postulate that the addition of rationally selected antibodies based on reports of their altered expression in SLE will result in an even higher discriminatory ability than currently used tests, with potential benefits both for diagnosis and monitoring of disease and tailoring of therapy.

## Supporting Information

Figure S1 **An image of the scanned microarray from a healthy control and SLE patients with different disease activity.**
(TIFF)

Figure S2 **Validation of expressed microarray CD antigen markers using flow cytometry.** **(A)** Unnormalized microarray spot intensity of four randomly selected expressed CD antigens in CD3 stimulated leukocytes. **(B)** Flow cytometry analysis of the same four antibodies in the same cell population, and also controls. All four measured expressed CD antigens detected by the microarray are also detected as being expressed by flow cytometry.
(TIFF)
