# Supplementary figures and images for: An Antibody-Based Leukocyte-Capture Microarray for the Diagnosis of Systemic Lupus Erythematosus
Source: PLoS One. 2013 Mar 13;8(3):e58199. doi: 10.1371/journal.pone.0058199 (PMC3596412; doi:10.1371/journal.pone.0058199)

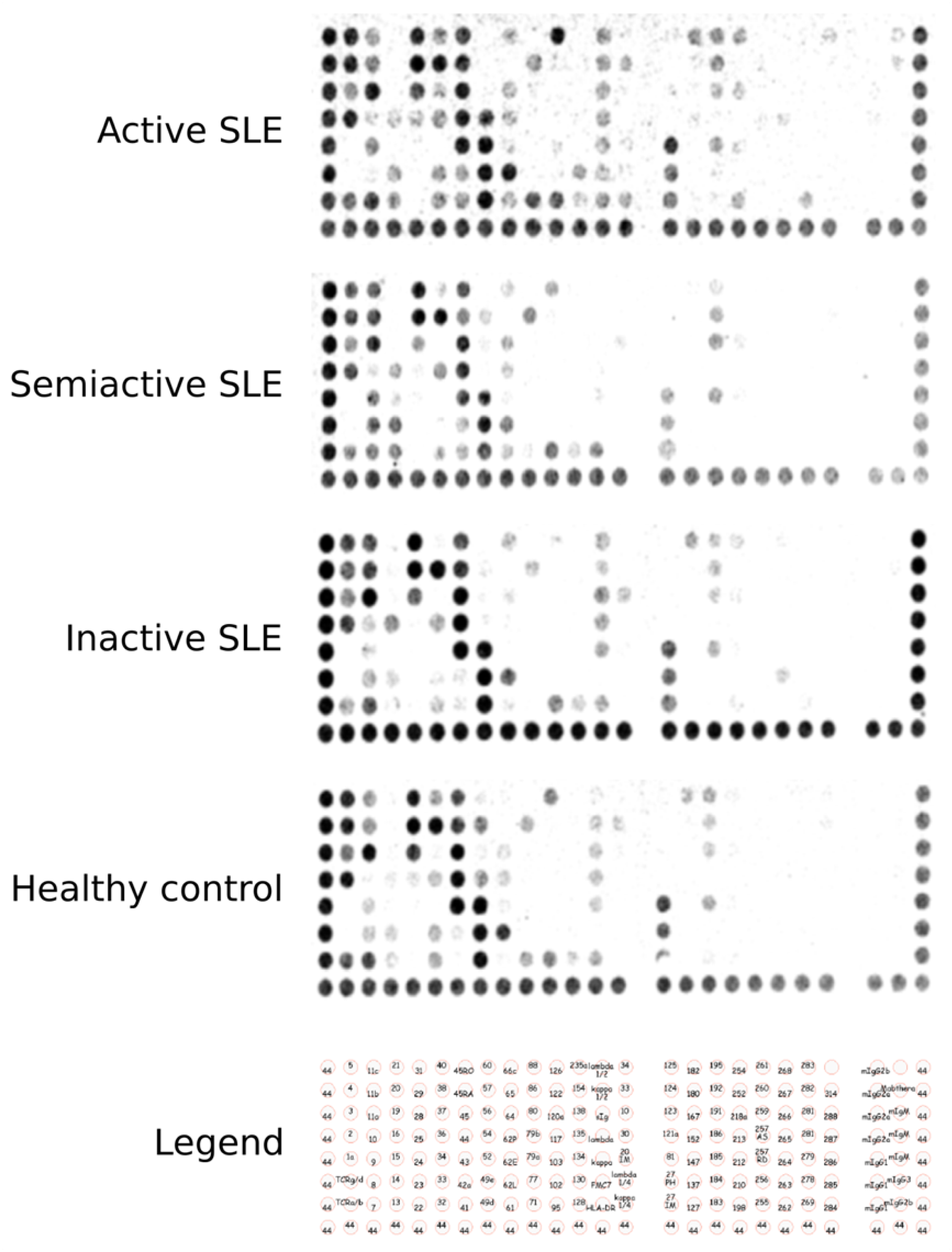

Supplement: Figure S1 — An image of the scanned microarray from a healthy control and SLE patients with different disease activity. (TIFF) [file pone.0058199.s001.tiff]

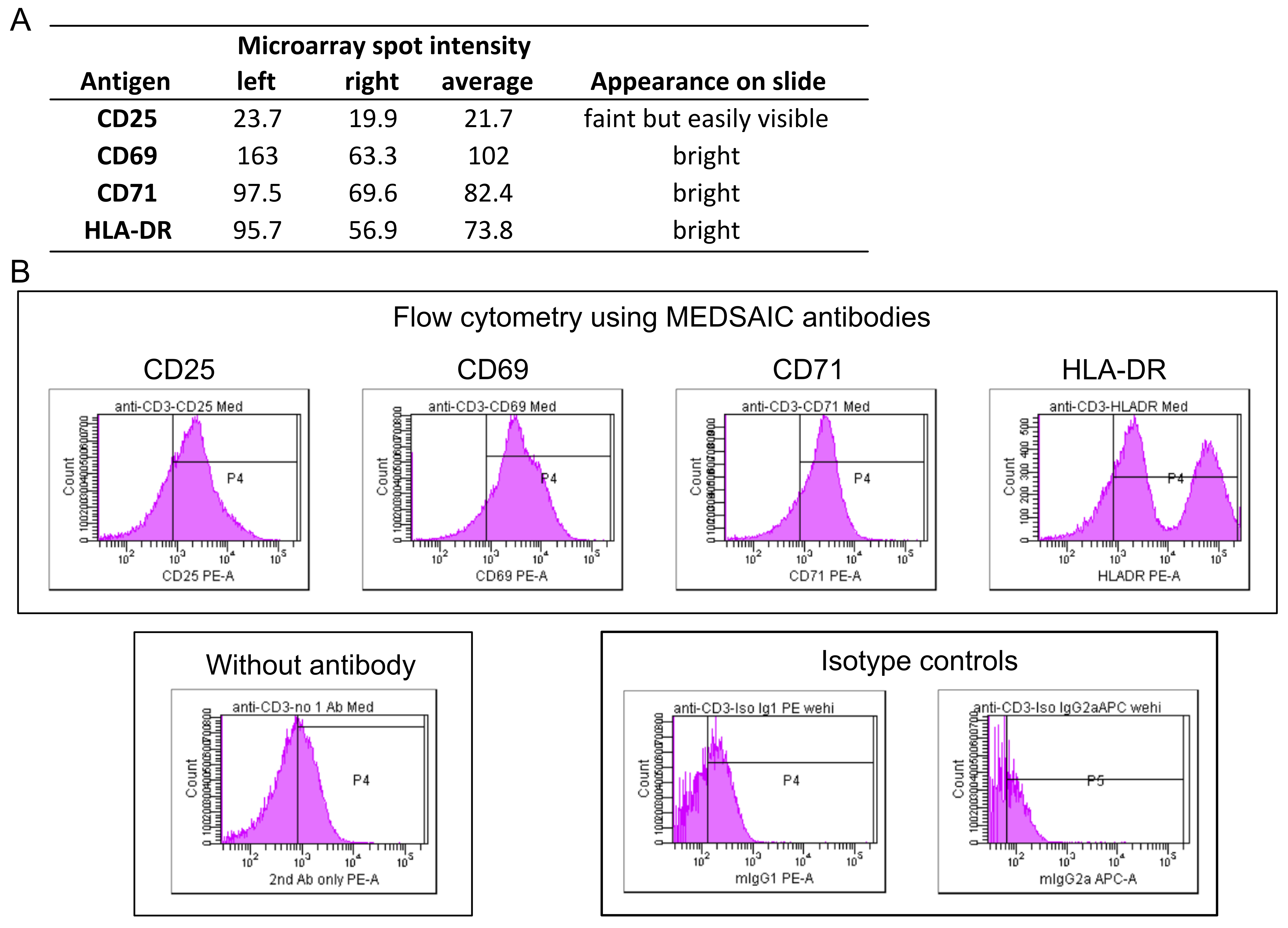

Supplement: Figure S2 — Validation of expressed microarray CD antigen markers using flow cytometry. (A) Unnormalized microarray spot intensity of four randomly selected expressed CD antigens in CD3 stimulated leukocytes. (B) Flow cytometry analysis of the same four antibodies in the same cell population, and also controls. All four measured expressed CD antigens detected by the microarray are also detected as being expressed by flow cytometry. (TIFF) [file pone.0058199.s002.tiff]
